# Prebiotic‐supplemented partially hydrolysed cow's milk formula for the prevention of eczema in high‐risk infants: a randomized controlled trial

**DOI:** 10.1111/all.12848

**Published:** 2016-02-26

**Authors:** R. J. Boyle, M. L.‐K. Tang, W. C. Chiang, M. C. Chua, I. Ismail, A. Nauta, J. O'B. Hourihane, P. Smith, M. Gold, J. Ziegler, J. Peake, P. Quinn, R. Rao, N. Brown, A. Rijnierse, J. Garssen, J. O. Warner, the PATCH study investigators, Christine Axelrad, Suzan Jeffries, Yvette Donald, Heather Barham, Jenny Brown, Rita Wickenden, Teresa Barnes, Simone Taylor, Susan Smith, Natalie Thomas, Anne Goh, Wong Anng Anng, Christy Cao Yu Hua, Deirdre Daly, Sinead Lafford, Claire Cullinane, Jacques Bindels, Liandre van der Merwe, Dineke Klaassen, Sophie Swinkels, Karen Knipping

**Affiliations:** ^1^ Section of PaediatricsImperial College LondonLondonUK; ^2^ Imperial College Healthcare NHS TrustLondonUK; ^3^ Royal Children's Hospital MelbourneMelbourneVic.Australia; ^4^ Murdoch Children's Research InstituteMelbourneVic.Australia; ^5^ University of MelbourneMelbourneVic.Australia; ^6^ KK Women's and Children's HospitalSingapore CitySingapore; ^7^ Nutricia ResearchUtrechtthe Netherlands; ^8^ Utrecht Institute for Pharmaceutical SciencesUtrechtthe Netherlands; ^9^ University CollegeCorkIreland; ^10^ Gold Coast HospitalGold CoastQldAustralia; ^11^ Women's and Children's HospitalAdelaideSAAustralia; ^12^ Sydney Children's HospitalSydneyNSWAustralia; ^13^ Royal Children's Hospital BrisbaneBrisbaneQldAustralia; ^14^ Poole Hospital NHS Foundation TrustPooleUK; ^15^ Salisbury Healthcare NHS TrustSalisburyUK

**Keywords:** eczema, infant formula, oligosaccharides, hydrolysate, randomized controlled trial

## Abstract

**Background:**

Prevention guidelines for infants at high risk of allergic disease recommend hydrolysed formula if formula is introduced before 6 months, but evidence is mixed. Adding specific oligosaccharides may improve outcomes.

**Objective:**

To evaluate whether partially hydrolysed whey formula containing oligosaccharides (0.8 g/100 ml) (pHF‐OS) can prevent eczema in high‐risk infants [ISRCTN65195597].

**Methods:**

We conducted a parallel‐group, multicentre, randomized double‐blind controlled trial of pHF‐OS *vs* standard cow's milk formula. Infants with a family history of allergic disease were randomized (stratified by centre/maternal allergy) to active (*n* = 432) or control (*n* = 431) formula until 6 months of age if formula was introduced before 18 weeks. Primary outcome was cumulative incidence of eczema by 12 months in infants randomized at 0–4 weeks (375 pHF‐OS, 383 control). Secondary outcomes were cumulative incidence of eczema by 12 or 18 months in all infants randomized, immune markers at 6 months and adverse events.

**Results:**

Eczema occurred by 12 months in 84/293 (28.7%) infants allocated to pHF‐OS at 0‐4 weeks of age, *vs* 93/324 (28.7%) control (OR 0.98 95% CI 0.68, 1.40; *P* = 0.90), and 107/347 (30.8%) pHF‐OS *vs* 112/370 (30.3%) control in all infants randomized (OR 0.99 95% CI 0.71, 1.37; *P* = 0.94). pHF‐OS did not change most immune markers including total/specific IgE; however, pHF‐OS reduced cow's milk‐specific IgG1 (*P* < 0.0001) and increased regulatory T‐cell and plasmacytoid dendritic cell percentages. There was no group difference in adverse events.

**Conclusion:**

pHF‐OS does not prevent eczema in the first year in high‐risk infants. The immunological changes found require confirmation in a separate cohort.

Breastfeeding is the optimal mode of infant feeding, and early formula introduction carries well‐documented risks to infant health and development 1, 2. Observational studies suggest that specific variations in early diet may be associated with allergic outcomes 3. Intervention trials in formula‐fed infants have found reduced eczema risk when formula is supplemented with a specific oligosaccharide mix 4, 5, 6. There is inconsistent evidence that partially hydrolysed formula reduces eczema risk compared with intact cow's milk formula in high‐risk infants 7, 8, 9. However, current dietary prevention guidance includes the use of partially or extensively hydrolysed formula for infants at an increased allergic disease risk where formula is introduced before 6 months 9, 10. Importantly, previous clinical studies have not evaluated markers of immune regulation, which may be important underlying mechanisms for preventing allergic disease. Exclusive breastfeeding rates are low in many countries, where early formula introduction is common 11. In the context of low breastfeeding rates and high allergic disease prevalence, the question whether partial hydrolysis of formula, oligosaccharide supplementation or both can prevent eczema is relevant.

We investigated whether a partially hydrolysed whey‐dominant formula supplemented with a specific oligosaccharide mixture (pHF‐OS) could reduce eczema cumulative incidence (as primary outcome) in infants at high risk of allergic disease who receive formula milk before age of 4 weeks. We also investigated whether the intervention could alter infant immune responses (as secondary outcomes), due to evidence that both pHF and oligosaccharides influence regulatory T cells 12, 13, 14.

## Methods

### Clinical study design and oversight

The study was a double‐blind, randomized, controlled parallel‐group nutritional intervention trial in infants at high risk of developing allergic disease, conducted in 10 specialist paediatric centres in Australia, Singapore, UK and Ireland from April 2006 to March 2011.

Singleton infants born ≥36 weeks of gestational age and ≥2500 g were eligible for participation in the study if at least one of their parents had a documented history of allergic disease, confirmed by means of skin prick testing or a history of anaphylaxis. Exclusion criteria were as follows: twins, infants with significant congenital abnormalities or severe neonatal illness, prematurity or low birth weight, consumption of cow's milk formula before randomization. Pregnant women at study centres were recruited in antenatal clinics or postnatal wards. After signed informed consent, women with or without their partner underwent skin prick testing if necessary, to confirm eligibility. Infants of eligible women were assessed soon after birth to confirm eligibility. Mothers were advised to exclusively breastfeed for 4–6 months according to regional/international guidance, and inclusion in the study was not dependent on mother's feeding intentions. The participant information sheet did not reveal the hypothesis that one type of infant formula may be more effective than another for preventing eczema. Study visits were conducted by a trained research nurse at 4, 8, 12, 18 weeks and 6, 12 and 18 months of age. Details of trial ethics approvals, monitoring and regulatory compliance are summarized in the Online Repository [study registration ISRCTN65195597 14th February 2006].

### Randomization and blinding

A trial statistician implemented a computer‐generated randomization sequence in blocks of 6, stratified by study centre and parental allergic history (maternal or dual heredity vs. only paternal). Mothers were advised to breastfeed their infants according to expert guidelines for allergy prevention and to contact the study team if they wished to initiate infant formula in the first 6 months. If this occurred before 18 weeks, infants were randomly allocated to active or control formula in a 1 : 1 ratio by the research nurse according to the randomization sequence. Active and control formulas were in coded tins identical in appearance, and the nurse accessed a code for each participant. Formula was labelled and coded by the clinical supply manager of Nutricia Research (previously Numico Research B.V. until November 2007; Danone Research until October 2013), who was not otherwise involved in the study. Participants, outcome assessors, research nurses and care providers remained blind to treatment allocation throughout the study. After the database was cleaned and locked, the treatment code was partially broken (treatment allocation X and Y), and after completion of all analyses described in the statistical analysis plan, the code was fully broken.

### Nutritional intervention

Assigned study formulae were delivered to participants at home or in hospital following randomization. If formula was introduced after 18 weeks (‘breastfed reference group’), infants were not randomized and received a commercially available formula of choice. Randomized infants received study formula until 6 months, manufactured and supplied by Nutricia Research, Cuijk, the Netherlands. The active group received a non‐ultra‐filtrated hydrolysed whey‐based infant formula to which a specific mixture of neutral scGOS and lcFOS (ratio 9 : 1; Immunofortis^®^, Nutricia Cuijk BV, Cuijk, the Netherlands; 85 weight per cent) and acidic pAOS (15 weight per cent) were added. Total oligosaccharide concentration was 0.8 g/100 ml (0.68 g/100 ml neutral; 0.12 g/100 ml acidic). The control group received a regular intact whey protein‐dominant infant formula without prebiotics. Both active and control formulae contained similar long‐chain polyunsaturated fatty acids.

### Primary and secondary outcome assessments

The primary outcome was cumulative incidence of eczema up to 12 months of age in infants who started formula before 4 weeks of age (‘early introduction subgroup’). Cumulative incidence of eczema up to 12 months of age in all infants randomized (‘all subjects randomized’) was a secondary outcome. Infants breastfed ≥18 weeks without formula comprised the breastfed reference group. Other secondary outcomes were time to eczema onset, eczema severity using SCORAD index at first presentation, gastrointestinal tract symptoms and immunological parameters including allergen‐specific serum immunoglobulins, regulatory T‐cell (Treg) and plasmacytoid dendritic cell (pDC) numbers and cytokine responses to allergen and mitogen. Stool biochemistry and faecal microbiota outcomes will be reported separately.

Eczema diagnosis was made by study physicians using modified Hanifin and Rajka criteria 15; severity and course of eczema was measured by trained study nurses using SCORAD at first presentation, 4 and 8 weeks later, then as needed. Stool frequency and consistency and gastrointestinal tract symptoms were assessed by parent‐completed questionnaires at all study visits using Likert scales 16. Blood was collected from randomized participants at 6 months for immune assessment.

### Immune parameters

Serum at 6 months was tested for total IgE, cow's milk‐ and hen's egg‐specific IgE and cow's milk‐ and hen's egg‐specific IgG1 and IgG4 using CAP‐FEIA and ELISA. Peripheral blood mononuclear cells (PBMCs) were collected from a subgroup of 85 infants. Eligible infants were those enrolled at a site able to process PBMCs (Singapore, Adelaide, Melbourne, London), with sufficient blood sample drawn to allow PBMC analysis in addition to serum analysis. PBMCs were cryopreserved for later batched analysis. Treg and dendritic cell populations were analysed by flow cytometry. PBMC culture supernatants were harvested at 48 h and analysed for the secretion of IFN‐γ, IL‐12, IL‐4, IL‐13, IL‐10, IL‐6, TNF‐α and TGF‐β after stimulation with PHA, TT, OVA or media alone by multiplex cytokine assay. Full details of immune laboratory methods are in the Online Repository.

### Data handling, statistical and interim analyses

Data were collected from paper Case Report Forms and entered into an electronic database by Clinquest Europe. Sample size: a reduction in cumulative incidence of eczema from 30% to 20% at 12 months was considered clinically relevant 17, 18, 19. Assuming 15% dropout, this study required 350 infants randomized before 4 weeks in each arm, to have 80% power to detect this reduction in eczema, using two‐tailed chi‐squared test at a significance of 0.05. The primary analysis data set was the ‘early introduction subgroup’, and secondary analyses were carried out on ‘all subjects randomized’ and the per protocol (PP) population. PP were ‘early introduction subgroup’ infants who continued study formula until 18 weeks, had not consumed intact cow's milk before 18 weeks and completed their 12‐month visit before 54 weeks. Outcomes were evaluated using logistic regression to generate OR, and Cox regression to generate HR, with 95% confidence intervals. Twelve‐ and 18‐month assessments were included if they occurred within a 2‐week window either side of the expected assessment date, for the analysis of primary outcomes, that is 52 ± 2 weeks and 78 ± 2 weeks. Secondary binary outcomes were also analysed using logistic regression; secondary continuous outcomes used multivariate linear regression unless stated otherwise. All clinical analyses were adjusted for specific stratification variables (i.e. centre, parental allergic history). Analyses were repeated including the prespecified covariates gender, ethnicity, mode of birth, household pet, siblings and birth weight. Analyses were performed by Public Health Sciences and Medical Statistics, University of Southampton, using SAS 9.3 (SAS Institute Inc, Cary, NC, USA). Planned interim analysis was conducted on safety and primary efficacy parameters, to evaluate safety and to verify whether assumptions underlying the sample size calculation were correct – these and statistical methods for immune outcomes are described in the Supporting information. Formal adjustment was not made for multiple analyses, because this can lead to overly conservative interpretation of findings, but statistical significance was interpreted cautiously where multiple analyses were made 20.

## Results

**Figure 1:**
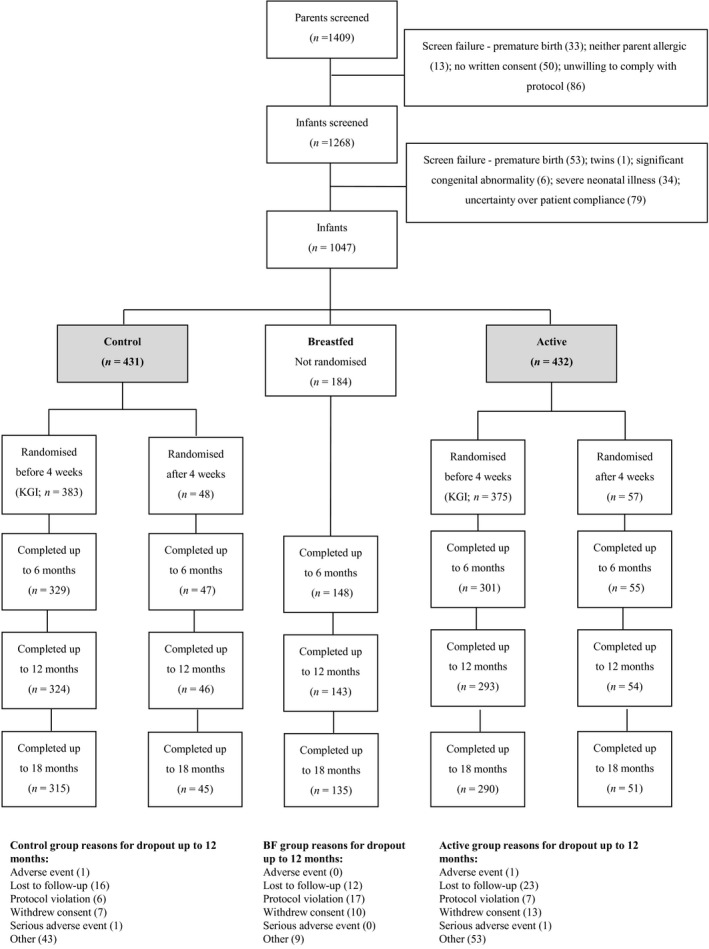
CONSORT flow diagram. Note some screen failures and losses to follow up included more than one reason.

In total, 1409 parents and 1268 infants were assessed for eligibility, and 1047 infants were enrolled (Fig. 1). Of these, 863 (82%) introduced formula prior to 18 weeks and were randomized to control or pHF‐OS formula (all subjects randomized), and 758 (72%) were randomized before 4 weeks (‘early introduction subgroup’). In the early introduction subgroup, 615 (81%) infants were assessed at 12 months and 594 (78%) at 18 months; in all subjects randomized, 714 (83%) and 688 (80%) respectively; in the breastfed group, 143 (78%) and 135 (73%). Participant characteristics are shown in Table 1. No clinically important differences between groups were observed at baseline, but early introduction of formula milk was more common in Singapore, where over half of randomized infants but less than 10% of breastfed infants were born. Cumulative incidence of eczema at 12 months also differed between centres, with lower rates in Singapore – 83/375 (22.1%) at 12 months; 89/369 (24.1%) 18 months – compared with all other centres – 94/242 (38.8%) at 12 months; 111/236 (47.0%) at 18 months. Parents of randomized infants (all subjects randomized) reported median formula intake of 570 mls per day (IQR 230, 750) at 4 weeks and 840 mls at 18 weeks (510, 1050).

**Table 1:** Baseline characteristics of PATCH study participants

	Control [ASR] (*N* = 431)	Control [Early*] (*N* = 383)	Active [ASR] (*N* = 432)	Active [Early*] (*N* = 375)	Breastfed (*N* = 184)
Maternal age (years)	30.0 (6.5)	29.7 (6.6)	29.3 (7.0)	29.0 (7.1)	31.6 (6.6)
Maternal tertiary education (%)	134 (31)	106 (28)	127 (29)	98 (26)	115 (63)
Gestational age (weeks)	38.9 (1.3)	38.8 (1.3)	39.0 (1.3)	38.9 (1.3)	39.7 (1.3)
Male sex (%)	209 (48)	185 (48)	238 (55)	204 (54)	104 (57)
Birth weight (g)	3306 (463)	3274 (462)	3302 (486)	3272 (481)	3541 (439)
Both parents allergic (%)	90 (21)	81 (21)	79 (18)	63 (17)	56 (30)
Only mother allergic (%)	240 (56)	215 (56)	250 (58)	224 (60)	94 (51)
Only father allergic (%)	99 (23)	85 (22)	102 (24)	87 (23)	34 (18)
Caucasian ethnicity (%)	157 (38)	118 (32)	156 (38)	113 (31)	150 (88)
Asian ethnicity (%)	240 (58)	237 (64)	237 (58)	234 (65)	10 (6)
Other ethnicity (%)	14 (4)	13 (4)	17 (4)	13 (4)	11 (6)
Vaginal delivery (%)	267 (62)	237 (62)	279 (65)	246 (66)	114 (62)
Instrumental delivery (%)	32 (7)	26 (7)	37 (9)	31 (8)	19 (10)
Caesarean delivery (%)	132 (31)	120 (31)	116 (27)	98 (26)	50 (27)
Pet at home (%)	130 (30)	111 (29)	138 (32)	110 (29)	58 (32)
At least one sibling (%)	235 (55)	206 (54)	231 (53)	203 (54)	91 (49)
Australia (%)	85 (20)	73 (19)	85 (20)	69 (18)	90 (49)
Ireland (%)	27 (6)	26 (7)	26 (6)	22 (6)	12 (7)
Singapore (%)	237 (55)	234 (61)	237 (55)	236 (63)	8 (4)
UK (%)	82 (19)	50 (13)	84 (19)	48 (13)	74 (40)

ASR, all subjects randomized; *Early, early introduction subgroup (infants who introduced formula milk early and were therefore randomized at <4 weeks of age). Continuous data are presented as mean (standard deviation).

### Effect of the intervention on cumulative incidence of eczema

**Figure 2:**
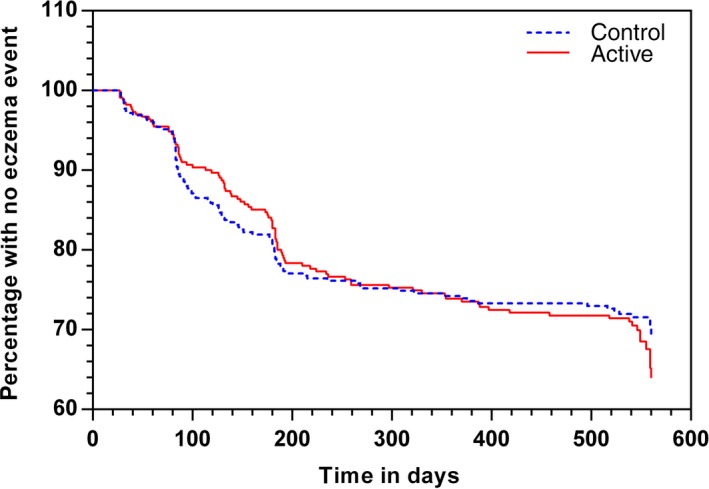
Kaplan–Meier plot of the time to first presentation of eczema in the group that was randomized before 4 weeks of age (‘early introduction subgroup’). There was no statistically significant difference between the groups (log–rank test: *P* = 0.81).

Eczema occurred by 12 months in 84/293 (28.7%) infants in the active group, *vs* 93/324 (28.7%) control in the ‘early introduction subgroup’ population (OR 0.98 95% CI 0.68, 1.40; *P* = 0.90; Table 2). Survival analysis using Cox regression showed no significant difference between groups in time to first onset of eczema (Fig. 2; *P* = 0.81). In all subjects randomized, eczema occurred by 12 months in 107/347 (30.8%) active, *vs* 112/370 (30.3%) control infants (OR 0.99 95% CI 0.71, 1.37; *P* = 0.94; Table 3). In both populations, there was also no significant difference in incidence of eczema by 18 months, in survival without eczema by 12 or 18 months, or in adjusted analyses for all predefined and significant covariates (Tables 2, 3). Findings were similar for the PP data set (Table S1). Eczema severity, expressed as median (IQR) SCORAD at the time of first diagnosis, did not differ in the active and control arm (14 (10, 23) and 14 (9, 23), respectively *P* = 0.97). In the ‘early introduction subgroup’ population, 21% with eczema had moderate–severe eczema (SCORAD ≥25) at the time of eczema diagnosis. In the breastfed reference group, 61/138 (44.2%) infants had eczema by 12 months and 63/136 (46.3%) by 18 months. History of allergic disease in both parents was present in 56/184 (30%) in the breastfed group *vs* 169/863 (20%) in randomized subjects.

**Table 2:** Effect of the intervention on incidence of eczema, in infants randomized prior to 4 weeks of age [‘early introduction subgroup’]

	Control	Active	Effect measure (95% CI)	Adjusted effect (95% CI) Model 1	Adjusted effect (95% CI) Model 2
Eczema by 12 months	93/324 (28.7%)	84/293 (28.7%)	OR 0.98 (0.68, 1.40) HR 1.04 (0.77, 1.40)	OR 0.94 (0.65, 1.36) HR 0.99 (0.73, 1.34)	OR 0.97 (0.67, 1.39) HR 1.03 (0.77, 1.39)
Eczema by 18 months	101/315 (32.1%)	99/290 (34.1%)	OR 0.88 (0.62, 1.25) HR 0.96 (0.73, 1.27)	OR 0.84 (0.59, 1.21) HR 0.92 (0.69, 1.23)	OR 0.88 (0.62, 1.25) HR 0.96 (0.73, 1.27)

All analyses were adjusted for stratification variables study centre and maternal allergic history. In addition, Model 1 adjusted for all predefined covariates (i.e. sex, ethnicity, mode of birth, pet exposure, presence of siblings, birth weight) and Model 2 adjusted for all covariates that were significant in Model 1 (birth weight at 12 months, none at 18 months).

**Table 3:** Effect of the intervention on incidence of eczema, in all infants randomized or not randomized (‘breastfed’ group)

	Control (%)	Active(%)	Effect measure (95% CI)	Adjusted effect (95% CI) Model 1	Adjusted effect (95% CI) Model 2	Breastfed group (%)
Eczema by 12 months	112/370 (30.3)	107/347 (30.8)	OR 0.99 (0.71, 1.37)	OR 0.94 (0.67, 1.32)	OR 0.97 (0.70, 1.35)	61/138 (44.2)
Eczema by 18 months	120/360 (33.3)	124/341 (36.4)	OR 0.88 (0.64, 1.22)	OR 0.84 (0.60, 1.17)	OR 0.88 (0.64, 1.22)	63/136 (46.3)

All analyses were adjusted for stratification variables study centre and maternal allergic history. In addition, Model 1 adjusted for all predefined covariates (i.e. sex, ethnicity, mode of birth, pet exposure, presence of siblings, birth weight) and Model 2 adjusted for all covariates that were significant in Model 1 (pet exposure at 12 months, none at 18 months).

### Effect of the intervention on infant immune development

#### Immunoglobulin levels

Serum from all subjects randomized with serum available was analysed for total IgE (*n* = 588), cow's milk‐specific IgE (*n* = 574), hen's egg‐specific IgE (*n* = 576), cow's milk‐specific IgG1 (*n* = 562) and hen's egg‐specific IgG1 (*n* = 547). Total and specific IgE levels did not differ between groups, as shown in Table 4. Specific IgE exceeded 0.35 kU/l in 16% for cow's milk and 14.5% for hen's egg at 6 months. Cow's milk‐specific IgG1 was significantly (*P* < 0.0001) lower in the active group – median 33.1 AU, IQR (10.0, 118.4), compared to control – median 825.8 AU, IQR (324.9, 1820.2). Hen's egg‐specific IgG1 did not differ between groups (Fig. S1). Total IgG1, IgG4, cow's milk‐ and hen's egg‐specific IgG4 did not differ between groups (data not shown).

**Table 4:** Effect of the intervention on serum immunoglobulin E profile in the group randomized before 4 weeks of age (‘early introduction subgroup’)

	Control	Active	*P* value^a^
Total IgE (kU/l)	9.01 (4.39, 18.60) [290]	8.55 (4.47, 16.90) [278]	0.51
Cow's milk‐specific IgE (kUa/l)	0.03 (0.01, 0.06) [292]	0.03 (0.01, 0.07) [282]	0.46
Hen's egg IgE (kUa/l)	0.01 (0.00, 0.03) [293]	0.00 (0.00, 0.02) [283]	0.15

Data are expressed as median (25th, 75th centile) [*n*].

a *P* values were calculated using the Mann–Whitney *U*‐test.

#### Plasmacytoid dendritic cells and regulatory T cells

**Figure 3:**
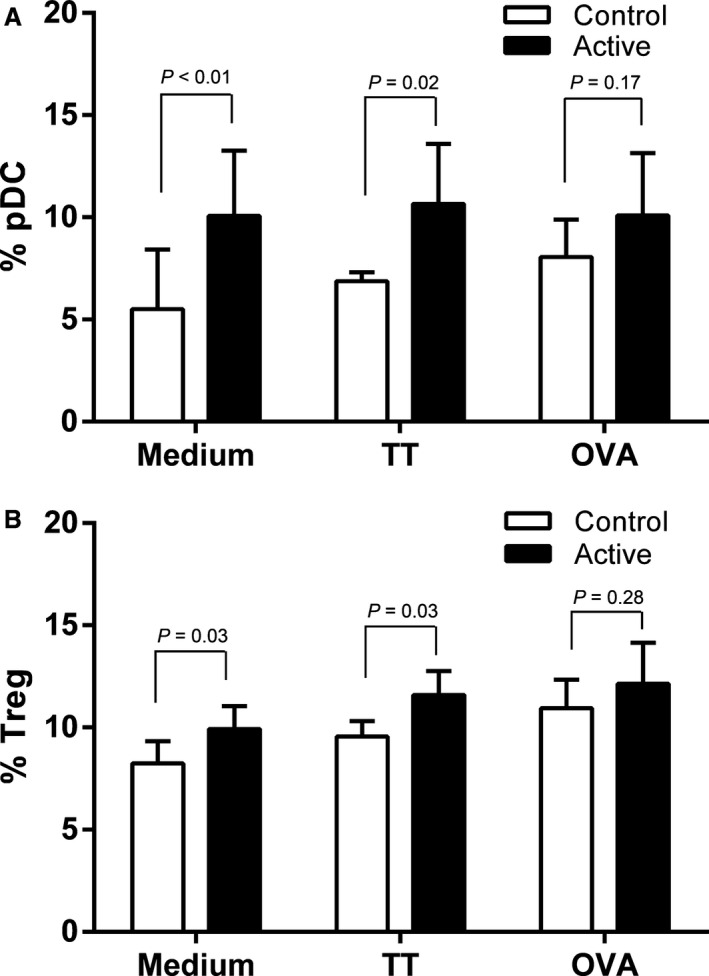
Plasmacytoid dendritic cell (pDC) and regulatory T‐cell (Treg) numbers in subjects receiving active or control formula (*n* = 92). The geometric mean percentage of all DCs that stained CD11c^lo^ CD123w^hi^ (A) and the geometric mean percentage of all CD4+ T cells that stained CD25^hi^FoxP3^hi^ (B) after 48‐h culture in medium, with tetanus toxoid (TT) or with ovalbumin (OVA), are shown. Bars are 95% confidence interval. *P* values are for two‐sided *t*‐test.

PBMCs were analysed from 85 infants (46 active and 39 control). Baseline characteristics of the PBMC subgroup and all subjects randomized are shown in Table S3. Infants in the active group had an increased CD11c^lo^CD123w^hi^ pDC percentage in unstimulated (*P* = 0.006) and tetanus toxoid‐stimulated PBMC (TT; *P* = 0.02), but not ovalbumin‐stimulated (OVA; *P* = 0.17) culture compared to the control group (Fig. 3A). No differences in mDC populations were observed for the three culture conditions (data not shown). Infants in the active group had increased CD4+CD25^hi^Foxp3^hi^ Treg percentage in PBMC cultured without stimulus (*P* = 0.03), with TT (*P* = 0.03), but not with OVA (*P* = 0.28; Fig. 3B).

#### PBMC cytokine responses

Cytokine levels were determined in cell culture supernatants of the PBMC subgroup. There were no significant differences in mean cytokine levels between active and control groups, except for IL‐4 (mean 0.29 pg/ml active; 0.08 pg/ml control; *P* = 0.001), IL‐13 (mean 1.45 pg/ml active; 0.58 pg/ml control; *P* = 0.06) and IFN‐ɣ (mean 0.33 pg/ml active; 0.13 pg/ml control; *P* = 0.054) in PBMC cultured with medium and for TNF‐α levels (mean 934.4 pg/ml active; 505.4 pg/ml control; *P* = 0.03) in culture with PHA.

### Adverse events

There was no significant difference between active and control groups in the number of infants with ≥1 adverse event (AE) or serious adverse event (SAE) (Table 5), or in total number of AE or SAE in specific categories (Tables S3 and S4). In the breastfed group, there were fewer participants with ≥1AE or SAE. Tables S5–S7 contain further details of specific adverse events, gastrointestinal tract symptoms and growth.

**Table 5:** Adverse events from enrolment to 18‐month follow‐up

	Control (*n* = 431) (%)	Active (*n* = 432) (%)	*P*	Breastfed (*n* = 184) (%)
No. participants with ≥1 adverse events	406 (94.2)	400 (92.6)	0.34	154 (83.7)
No. with ≥1 related adverse event	130 (30.2)	114 (26.4)	0.22	–
No. with ≥1 serious adverse event	92 (21.3)	81 (18.8)	0.34	19 (10.3)
No. with ≥1 related serious adverse event	2 (0.5)	8 (1.9)	0.11	–

## Discussion

### Main findings

In this multicentre international randomized controlled trial, we found no evidence that a partially hydrolysed, whey‐dominant infant formula, supplemented with a specific oligosaccharide mixture, reduced incidence of eczema at 12 and 18 months in high‐risk infants, compared with standard cow's milk formula. These findings contrast with some previous studies suggesting that hydrolysed formula or prebiotics alone may reduce eczema risk in formula‐fed infants 4, 5, 6, 7. We also found no effect on total or specific IgE levels, or a range of other immune markers at 6 months. We did find some positive immune effects; however because no adjustments have been made for multiple comparisons, these immune findings should be interpreted with caution. We found that infants in the intervention group had significantly lower levels of IgG1 against cow's milk and increased percentages of Treg and pDC in both unstimulated and tetanus toxoid‐stimulated cultures as compared to control infants. This supports the concept that infant dietary exposures may influence immune development, although these specific changes require confirmation in a separate cohort and their relevance to longer‐term health outcomes is not yet clear.

### Comparison with other studies

The effect of hydrolysed infant formula intake on eczema risk is controversial, with positive findings in one large trial and negative findings in a separate large trial 7, 8. Consistent with the negative study, our data failed to demonstrate a protective effect of partially hydrolysed prebiotic‐supplemented infant formula against eczema incidence. Two previous trials evaluated an oligosaccharide mixture for eczema prevention, one comparing a standard cow's milk formula with or without the same prebiotics as our study, but in a low risk cohort 4, one comparing an extensively hydrolysed formula with or without prebiotics in a high‐risk population. The two studies found similar reductions in eczema incidence over the first year of life in the prebiotic‐supplemented groups 4, 5, 6. There are differences between our trial and the previous successful prebiotic trials. First was the use of partially hydrolysed formula, which itself may have immune effects and therefore interact with prebiotic effects and this may alter any effect of prebiotics on eczema risk 14. If the impact of prebiotics is primarily on moderation of the immune response to dietary antigens, it may be more appropriate to combine exposure to nonhydrolysed proteins to achieve optimal long‐term benefit. Second, this study included non‐European study sites with very low breastfeeding rates and other environmental and genetic differences – however, our findings were similar across sites, suggesting that inclusion of non‐European study sites cannot explain the discrepant findings alone. Overall, our data cast doubt on the concept of hypo‐allergenicity through hydrolysis of infant formula, and the role of prebiotics, at least in relation to influencing eczema.

While early feeding with pHF‐OS was associated with reduced cow's milk IgG1 at 6 months, no effect on OVA IgG1 or any allergen‐specific IgE was observed. Although IgE is considered to be the key immunoglobulin in the pathogenesis of allergic sensitization and allergic diseases, early food‐specific IgG1 has also been found to correlate with later development of allergic disorders. Children who were sensitized 21 or asthmatic 22 at 5 years had higher hen's egg‐specific IgG1 in early life. In addition, high‐risk infants with high food‐specific IgG1 were more likely to develop inhalant‐specific IgE, compared with subjects with low food‐specific IgG1 21, 23, 24. These findings suggest that raised food‐specific IgG1 could represent a predictive biomarker for future allergic disease, in high‐risk infants. This may have been mediated by the hydrolysis rather than prebiotics because hen's egg‐specific IgG1 was not altered, and others have also reported that hydrolysis can reduce cow's milk‐specific IgG1 25. The increased pDC and Treg percentage in infants receiving pHF‐OS is consistent with increased tolerogenic immune responses, as both cell types play an important role in programming and regulating immune responses 26 and reducing the risk of Th2‐mediated allergic immune responses 27. While it is not possible to distinguish the individual effects of hydrolysate and prebiotic, it is possible that both components contributed to the observed immune effects. In a mouse model of cow's milk allergy, hydrolysed formula feeding increased mesenteric lymph node suppressor Foxp3^+^ Treg cell number that could confer protective effects to naïve recipient mice 14. The specific oligosaccharide mixture used in our study was shown to modulate the CD25^+^ T regulatory cell compartment in mice 12, 13. Overall, the nature and relevance of the immune changes seen requires confirmation in a separate population.

### Strengths and limitations

The concept of hypo‐allergenicity in relation to infant milk formulae should now be re‐evaluated. Recent insights indicate that the primary abnormality in eczema is a skin barrier defect. Allergic sensitization is a secondary phenomenon which increases the severity and longevity of atopic disorders 28. Thus, interventions that reduce sensitization and/or induce tolerance may be more important for long‐term outcomes. Our study showed that the pHF‐OS did not reduce early eczema, but suggested that pHF‐OS was hypo‐antigenic and had immune modulatory effects including increased regulatory T‐cell numbers which may protect against risk of later allergic diseases such as asthma and allergic rhinitis. Further studies are now required to confirm these immune findings and to establish whether they are associated with longer‐term health implications.

The strength of our study is that of a well‐conducted international multicentre trial, testing a pragmatic intervention. The disadvantage of combining two different interventions in one treatment arm is that we are not able to distinguish separate effects for each intervention (partially hydrolysed formula and prebiotic supplementation) – it is therefore possible, albeit unlikely, that the interventions interacted to lead to no clinical effect, whereas separate use of one of the interventions might have resulted in a different outcome. Our analyses of PBMC phenotype and function were limited by relatively small numbers, and findings must be interpreted cautiously as they were not corrected for multiple comparisons, and the study set‐up necessitated the use of cryopreserved cells which can influence PBMC phenotype and function 29. Our study took place in a variety of settings in different countries, so the findings are likely to be generalizable to a range of settings. They may not be generalizable to infants without a family history of allergic disease and are not relevant to populations or individuals who do not use cow's milk‐based infant formula in the first 18 weeks of life.

## Conclusions

In summary, we found no evidence that a partially hydrolysed formula containing a specific mixture of oligosaccharides influences eczema incidence to 18 months, or total/specific IgE at 6 months, in high‐risk infants fed a cow's milk‐based formula milk in the first 18 weeks of life. These findings contrast with some previous studies which found reduced eczema in infants fed partially hydrolysed formula or prebiotics alone. Studies in new populations are needed to confirm the lower levels of CM‐IgG1 and increase in Treg and pDC seen in this study, and establish whether they are relevant to longer‐term health outcomes.

## Conflict of interest

The study was funded by Nutricia Research. MLKT is a member of the ANZ medical advisory boards for Danone Nutricia and Nestle Nutrition Institute; Global scientific advisory board for Danone Nutricia; and has received honoraria for presentations at symposia sponsored by Danone Nutricia and Nestle Nutrition Institute. JOBH is chair of the Irish Food Allergy Network which receives unrestricted educational grants from Danone Nutricia and other manufacturers of infant formulae and has received honoraria for presentations at symposia sponsored by Danone Nutricia and other companies. JOW is a member of the global advisory board for Danone Nutricia and has received grants and honoraria for presentations at symposia sponsored by Danone Nutricia. The other authors declare no conflict of interest.

## Author contributions

JOW designed the study and oversaw implementation of the study, analysis and interpretation of findings. RJB, MLKT and AN contributed to the design of secondary immunological analyses. II undertook immunological assays, and MLKT and AN led the analysis of these data. RJB, MLKT, WCC, MCC, JOBH, PS, MG, JZ, JP, PQ, RR and NB oversaw recruitment, treatment allocation, follow‐up and data collection at their study site. AR and JG supported study design and interpretation. RJB and MLKT wrote the manuscript. All authors contributed to the interpretation of findings and write‐up of the manuscript.

## Supporting information


**Data S1** Methods

**Figure S1** Serum levels of specific immunoglobulin G subclass 1 (IgG1) for hen's egg (control *n* = 281, active *n* = 266) and cow's milk (control *n* = 285, active *n* = 277) at 6 months of age in the group that was randomised before 4 weeks of age (‘early introduction subgroup’).

**Table S1**. Effect of the intervention on incidence of AD, in per protocol analysis
**Table S2.** Baseline characteristics of all randomised subjects and the subgroup selected for PBMC analysis
**Table S3**. Adverse Events from enrolment to 18 months follow up – specific categories
**Table S4**. Serious Adverse Events from enrolment to 18 months follow up – specific categories
**Table S5**. Gastrointestinal symptoms in study participants
**Table S6**. Stool frequency and consistency in study participants
**Table S7**. Growth measures in study participants randomised before 4 weeks age or not randomised (‘breastfed’).

## Appendix 1 PATCH Study Team

Christine Axelrad, Royal Children's Hospital Melbourne; Suzan Jeffries, Imperial College London; Yvette Donald and Heather Barham, Poole Hospital; Jenny Brown and Rita Wickenden, Salisbury District Hospital; Teresa Barnes, Gold Coast Hospital; Simone Taylor, Royal Children's Hospital Brisbane; Susan Smith, Sydney Children's Hospital; Natalie Thomas, Women's and Children's Hospital Adelaide; Anne Goh, Wong Anng Anng and Christy Cao Yu Hua, KK Women's and Children Hospital Singapore; Deirdre Daly, Sinead Lafford and Claire Cullinane, Cork University Hospital; Jacques Bindels, Liandre van der Merwe, Dineke Klaassen, Sophie Swinkels and Karen Knipping, Nutricia Research.
