# Supplementary material for: Prebiotic‐supplemented partially hydrolysed cow's milk formula for the prevention of eczema in high‐risk infants: a randomized controlled trial
Source: Allergy. 2016 Feb 26;71(5):701–10. doi: 10.1111/all.12848 (PMC4996326; doi:10.1111/all.12848)
Supplement: Supplementary file 1 — Data S1 Methods [file ALL-71-701-s001.docx]

**Prebiotic-supplemented partially hydrolysed cow’s milk formula for the prevention of eczema in high risk infants: a randomised controlled trial**

R. J. Boyle^1,2*^, M. L-K. Tang^3,4,5*^, W. C. Chiang^6^, M. C. Chua^6^, I. Ismail^4,5^, A. Nauta^7,8^, J. O’B. Hourihane^9^, P. Smith^10^, M. Gold^11^, J. Ziegler^12^, J. Peake^13^, P. Quinn^11^, R. Rao^14^, N. Brown^15^, A. Rijnierse^7,8^, J. Garssen^7,8^, J. O. Warner^1,2^ on behalf of the PATCH study investigators†

^1^Section of Paediatrics, Imperial College London, UK; ^2^Imperial College Healthcare NHS Trust, London UK; ^3^Royal Children’s Hospital Melbourne; ^4^Murdoch Children’s Research Institute; ^5^University of Melbourne, Australia; ^6^KK Women’s and Children’s Hospital, Singapore; ^7^Nutricia Research, Utrecht, the Netherlands; ^8^Utrecht Institute for Pharmaceutical Sciences, Utrecht, the Netherlands; ^9^University College, Cork, Ireland; ^10^Gold Coast Hospital, Gold Coast, Australia; ^11^Women’s and Children’s Hospital, Adelaide, Australia; ^12^Sydney Children’s Hospital, Sydney, Australia; ^13^Royal Children’s Hospital Brisbane, Brisbane, Australia; ^14^Poole Hospital NHS Foundation Trust, Poole, UK; ^15^Salisbury Healthcare NHS Trust, Salisbury, UK

**Corresponding author:**

Professor John Warner

Department of Paediatrics

Imperial College London

Norfolk Place, London UK. W2 1PG

Tel: +44 207 594 3990 Fax: +44 207 594 3984

email: [j.o.warner@imperial.ac.uk](mailto:j.o.warner@imperial.ac.uk)

*Both authors contributed equally

**Conflict of interest**: The study was funded by Nutricia Research. MLKT is a member of the ANZ medical advisory boards for Danone Nutricia and Nestle Nutrition Institute; Global scientific advisory board for Danone Nutricia; and has received honoraria for presentations at symposia sponsored by Danone Nutricia and Nestle Nutrition Institute.  JOBH is chair of the Irish Food Allergy Network which receives unrestricted educational grants from Danone Nutricia and other manufacturers of infant formulae and has received honoraria for presentations at symposia sponsored by Danone Nutricia and other companies. JOW is a member of the global advisory board for Danone Nutricia and has received grants and honoraria for presentations at symposia sponsored by Danone Nutricia. The other authors declare no conflict of interest.

**†PATCH Study Team:** Christine Axelrad, Royal Children’s Hospital Melbourne; Suzan Jeffries, Imperial College London; Yvette Donald and Heather Barham, Poole Hospital; Jenny Brown and Rita Wickenden, Salisbury District Hospital; Teresa Barnes, Gold Coast Hospital; Simone Taylor, Royal Children’s Hospital Brisbane; Susan Smith, Sydney Children’s Hospital; Natalie Thomas, Women’s and Children’s Hospital Adelaide; Anne Goh, Wong Anng Anng and Christy Cao Yu Hua, KK Women’s and Children Hospital Singapore; Deirdre Daly, Sinead Lafford and Claire Cullinane, Cork University Hospital; Jacques Bindels, Liandre van der Merwe, Dineke Klaassen, Sophie Swinkels and Karen Knipping, Nutricia Research.

**Keywords:**

Partial hydrolysate, infant formula, dietary intervention, oligosaccharides, randomised controlled trial, eczema, immune system, prebiotics

**Methods**

**Ethics approval, monitoring and regulatory compliance**

The trial observed International Conference on Harmonisation-Good Clinical Practice (ICH-GCP) guidelines, the Declaration of Helsinki and applicable local ethical and legal requirements. An independent Data Monitoring Committee was established to perform safety surveillance and to re-evaluate sample size assumptions. Ethical approval was obtained from the Southampton & South West Hampshire Research Ethics Committee (Central Ethics Committee, England), Clinical Research Ethics Committee Of The Cork Teaching Hospitals (Ireland), SingHealth Centralised Institutional Review Board E (Singapore), Gold Coast Health Service District Human Research Ethics Committee, CYWHS Human Research Ethics Committee, Royal Children’s Hospital Melbourne, Ethics and Research Office, Ethics in Human Research Committee of Royal Brisbane and Women's Hospital, and Human Research Ethics Committee – Northern Network (Australia). The study was monitored throughout by the study sponsor, to ensure ICH-GCP compliance. Investigational products, accountability logs and storage facilities were monitored.

**Safety monitoring and growth assessment**

Number, type and severity of adverse and serious adverse events reported by investigators or parents were recorded throughout the study. For safety monitoring, plasma nutritional/protein indicators (total protein, albumin, urea, plasma amino-acids) were investigated in a subgroup (n=34) to inform the interim analysis. Anthropometric measurements were taken in duplicate at baseline and 4 and 12 weeks, and 6, 12 and 18 months. Weight was measured (unclothed) to the nearest 0.1kg using an electronic weighing scale. Length was measured on a length board to the nearest 0.1 cm, and head circumference around the maximum circumference of the head (forehead to occiput) to the nearest 0.1 cm using a stretch-proof measuring tape.

**Preparation of blood samples for analysis of mononuclear cells**

Blood samples were collected at 6 months. Peripheral blood mononuclear cells (PBMC) were separated using Lymphoprep (Axis-Shield, UK) gradient centrifugation and cryopreserved at 8–10 x10^6^ cells/mL in RPMI with 42.5% foetal calf serum (FCS) and 7.5% dimethyl sulfoxide (DMSO) (Gibco, Grand Island, NY, USA) for subsequent batched analyses. The cryopreservation method at relevant participating sites was validated prior to collection of samples from subjects to ensure optimal retrieval and viability of PBMC with cryopreservation and thaw.

**Serum immunoglobulin analysis**

Serum samples were stored at -20°C until testing. Total IgE (n=568), specific IgE and IgG4 against cow’s milk (n=574), hen’s egg (ovalbumin (ova); n=576) were determined using the Immunocap**®** system (Thermo-Fisher, Uppsala, Sweden). Specific IgG1 against cow’s milk (n=562) and hen’s egg (n=547) was analysed by an in house ELISA. The detection limit for total IgE was 2 kU/L, for specific IgE 0.01 kU/L and for cow’s milk IgG1 1.45 AU and for hen’s egg IgG1 2.4 AU.

**PBMC culture**

Cryopreserved mononuclear cells were thawed and cultured as described previously ([12](#_ENREF_12)). In brief, thawed PBMC were cultured at 1x10^6^ cells/mL in AIM-V serum-free medium (Gibco, Grand Island, USA) supplemented with 2-mercaptoethanol (4x10^-5^ M; Gibco, Grand Island, USA) and stimulated with PHA (1 μg/ml; Fisher Scientific, Loughborough, UK), OVA (100 μg/ml; Sigma, St Louis, MO, USA) or tetanus toxoid (1 μg/ml; Statens Serum Institut, Copenhagen , Denmark), or without stimulus (medium alone) for 48 hours for measurement of cytokine production and dendritic cell analyses. Parallel IFN-γ/LPS culture (to assess IL-12 production) was performed by priming PBMC (at 5x10^6^ cells/mL) with recombinant IFN-γ (10 μg/ml; Boehringer Ingelheim, Germany) for 3 hours followed by stimulation with LPS (10 μg/ml; Sigma, St Louis, MO, USA) in Roslyn Park Memorial Institute (RPMI) (Gibco, Grand Island, NY, USA) supplemented with 10% foetal calf serum (FCS) culture medium.

**Flow cytometry**

Cell pellets were stained with fluorochrome-conjugated monoclonal antibodies or isotype controls in 50 µL staining volume. For enumeration of dendritic cell (DC) subsets, cells were stained with lineage cocktail–fluorescein isothiocyanate (FITC) (anti-CD3, 14, 16, 19, 20, 56), HLA-DR–peridinin chlorophyll protein (PerCP), CD123w–phycoerythrin (PE) and CD11c–allophycocyanin (APC). DC phenotypes were identified as CD11c^hi^CD123w^lo^ myeloid DC (mDC), CD123^hi^CD11c^lo^ plasmacytoid DC (pDC) and CD11c^lo^CD123w^lo^ immature DC (iDC). For assessment of Tregs, cell pellets were stained with CD3-APC, CD4- PerCP, and CD25-PE-CY7, followed by intracellular staining with forkhead box P3 (FoxP3)-PE to identify FoxP3hiCD25hi CD4+ Treg cell populations. Antibodies used were all from BD Bioscience (San Jose, CA, USA), except for CD25-PE-CY7 and FoxP3-PE from e-Bioscience (San Diego, CA, USA). For intracellular staining, cells were subsequently permeabilised, fixed and stained with FoxP3-PE antibody according to the manufacturer’s instructions (e-Bioscience, San Diego, CA, USA). Fluorescence data were acquired on a 4-colour LSR II (BD, San Jose, CA) and analysed with FACSDiva v4.1 software using well-defined gating strategies.

**Cytokine analysis**

Cell-free supernatants were collected at 48 hours (24 hours for IL-12p40) and stored at -20°C. Concentrations of IFN-γ, IL-4, IL-13, IL-10, IL-6, and TNF-α were determined by multiplex cytokine bead assay using a Luminex 100 Analyser (Luminex Corporation, Austin, TX, USA). Anti-cytokine beads and matched anti-cytokine biotinylated reporters were used according to the manufacturer’s instructions (Millipore, Billerica, MA, USA). Data were analysed with Luminex IS 2.3 software using a five-parameter regression formula to calculate sample concentrations from the standard curves and expressed in pg/mL. TGF-β1 and IL-12p40 were assayed by commercial human TGF-β1 and IL-12p40 ELISA kits according to the manufacturer’s protocol (R&D Systems, Minneapolis, USA). TGF-β1 and IL-12p40 concentrations were determined based on a standard curve generated by KCjunior v1.40.3 program (Bio-Tek Instruments, Winooski, VT, USA) using a 4 parameter equation and results reported in pg/mL. All supernatants were analysed undiluted in duplicate.

**Interim statistical analysis**

To verify whether the assumptions underlying the sample size calculation were correct and to evaluate safety, an interim analysis was performed in December 2008 on safety and primary efficacy parameters. The interim analysis was based on 141 randomised subjects and 39 breastfed subjects that reached the age of 12 months. The independent trial statistician presented the (independent) DMC with semi-blinded cumulative incidence of eczema, a list of all safety data, and any data requested by the DMC statistician. The DMC did not find any significant safety concerns. They found a 21% cumulative incidence rate of eczema at 12 months, which was lower in Singapore than other centres. Their final recommendation was to continue the study. However, it was advised to balance the ratio Asian and Caucasian subjects. To do so, it was decided to stop inclusion in Singapore prior to what was initially agreed, and to continue inclusion in the United Kingdom (UK) and Australia.

**Analysis of immunological outcomes**

For the sub study on PBMC stimulation, a sample size of 50 in each arm was required to provide 90% power to detect a 1.5-fold or greater difference between groups for mean Treg populations (as a proportion of all CD4+cells) or pDC (as a % of all DC). Data were generally not normally distributed; some skewed data were log_10_ transformed and others could not be transformed to normality. The Student’s t-test was used to analyse normally distributed continuous data, and Mann–Whitney U-test was used for skewed data. Data were presented as either geometric means with 95% CI, or median with interquartile range (IQR) depending on the distribution. All statistical analyses were performed using SAS software. A P-value of <0.05 was considered statistically significant for all analyses.

**Participating centres**

Participating study centres were Imperial College London, London, St. Mary’s Hospital, Poole Hospital NHS Trust, Poole, and Salisbury Healthcare NHS Trust, Salisbury, United Kingdom; Cork University Hospitals, Cork, Ireland, KK Women’s and Children’s Hospital, Singapore, Gold Coast Hospital, Gold Coast, Royal Children’s Hospital Brisbane, Brisbane, Sydney Children’s Hospital, Sydney, Women’s and Children’s Hospital, Adelaide, and Royal Children’s Hospital, Melbourne, Australia. The institutional ethics committees of all study centres approved the study protocol.

**Data Monitoring Committee**

Prof. H. Van Bever, Chairman (National University Hospital, Singapore)

Prof. U. Wahn, Paediatrician (Charite Hospital, Berlin)

Dr. R. W. Meyer, Dietician (Imperial College London, United Kingdom)

Prof. Dr. J.W.R. Twisk, Biostatistician (Vrije Universiteit Medisch Centrum, Netherlands)

**Results**

**Safety, growth and gastrointestinal symptoms**

The specific categories of adverse event with reduced numbers in the breastfed group were ‘pregnancy, puerperium, perinatal conditions’ for AE, and ‘infections and infestations’ for both AE and SAE. In the category ‘surgical and medical circumstances’ there were more AE in the breastfed than formula fed groups. Gastrointestinal symptoms were recorded in more detail than other potential adverse events. There was a lower prevalence of frequent burping during (but not after) the intervention period in the active group (P=0.048; Table S5) but other gastrointestinal symptoms did not differ significantly between groups. Stool frequency was increased in the active group at 4 weeks and 6 months, and stools were more watery at 12 weeks but harder at 1 year in the active group (Table S6). Active treatment was associated with increased length and head circumference at 4 and 12 weeks, but no differences in growth parameters were observed in active and control groups beyond the treatment period (Table S7). Breastfed infants had increased stool frequency and increased watery stools, and were larger than formula fed infants from prior to the intervention period onwards.

**Figure Legends**

Figure S1. Serum levels of specific immunoglobulin G subclass 1 (IgG1) for hen’s egg (control n=281, active n=266) and cow’s milk (control n=285, active n=277) at 6 months of age in the group that was randomised before 4 weeks of age (‘early introduction subgroup’). Data are median and IQR. P values are for two-sided t-test with Welch’s correction.
