# Supplementary figures and images for: Prebiotic‐supplemented partially hydrolysed cow's milk formula for the prevention of eczema in high‐risk infants: a randomized controlled trial
Source: Allergy. 2016 Feb 26;71(5):701–10. doi: 10.1111/all.12848 (PMC4996326; doi:10.1111/all.12848)

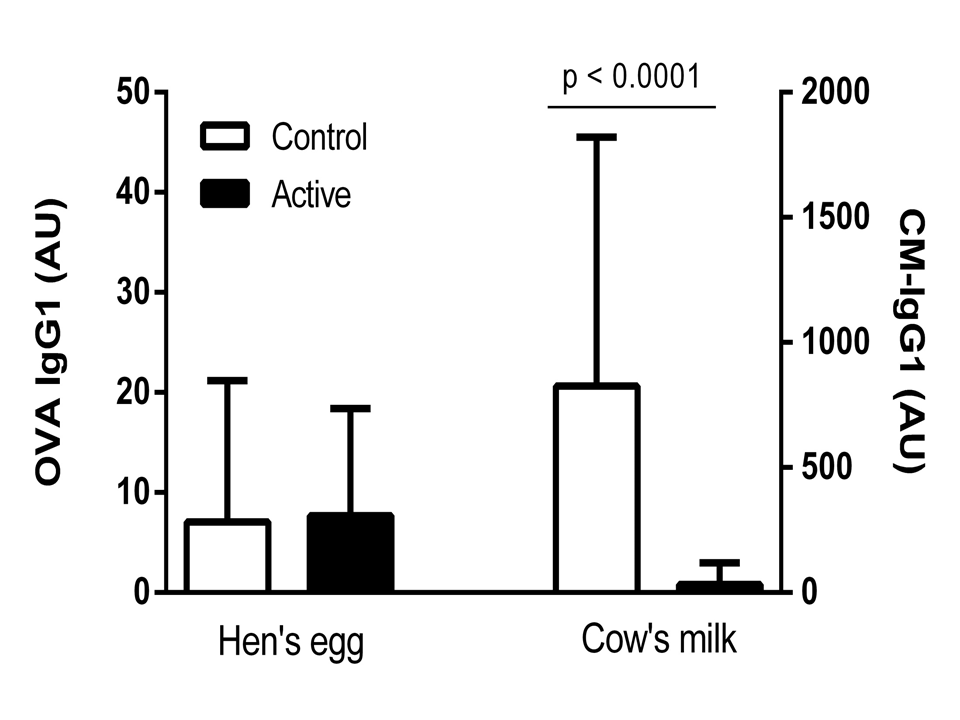

Supplement: Supplementary file 2 — Figure S1 Serum levels of specific immunoglobulin G subclass 1 (IgG1) for hen's egg (control n = 281, active n = 266) and cow's milk (control n = 285, active n = 277) at 6 months of age in the group that was randomised before 4 weeks of age (‘early introduction subgroup’). [file ALL-71-701-s002.tif]
