# Supplementary material for: Prebiotic‐supplemented partially hydrolysed cow's milk formula for the prevention of eczema in high‐risk infants: a randomized controlled trial
Source: Allergy. 2016 Feb 26;71(5):701–10. doi: 10.1111/all.12848 (PMC4996326; doi:10.1111/all.12848)
Supplement: Supplementary file 3 — Table S1. Effect of the intervention on incidence of AD, in per protocol analysis Table S2. Baseline characteristics of all randomised subjects and the subgroup selected for PBMC analysis Table S3. Adverse Events from enrolment to 18 months follow up – specific categories Table S4. Serious Adverse Events from enrolment to 18 months follow up – specific categories Table S5. Gastrointestinal symptoms in study participants Table S6. Stool frequency and consistency in study participants Table S7. Growth measures in study participants randomised before 4 weeks age or not randomised (‘breastfed’). [file ALL-71-701-s003.docx]

**Prebiotic-supplemented partially hydrolysed cow’s milk formula for the prevention of eczema in high risk infants: a randomised controlled trial**

R. J. Boyle^1,2*^, M. L-K. Tang^3,4,5*^, W. C. Chiang^6^, M. C. Chua^6^, I. Ismail^4,5^, A. Nauta^7,8^, J. O’B. Hourihane^9^, P. Smith^10^, M. Gold^11^, J. Ziegler^12^, J. Peake^13^, P. Quinn^11^, R. Rao^14^, N. Brown^15^, A. Rijnierse^7,8^, J. Garssen^7,8^, J. O. Warner^1,2^ on behalf of the PATCH study investigators†

^1^Section of Paediatrics, Imperial College London, UK; ^2^Imperial College Healthcare NHS Trust, London UK; ^3^Royal Children’s Hospital Melbourne; ^4^Murdoch Children’s Research Institute; ^5^University of Melbourne, Australia; ^6^KK Women’s and Children’s Hospital, Singapore; ^7^Nutricia Research, Utrecht, the Netherlands; ^8^Utrecht Institute for Pharmaceutical Sciences, Utrecht, the Netherlands; ^9^University College, Cork, Ireland; ^10^Gold Coast Hospital, Gold Coast, Australia; ^11^Women’s and Children’s Hospital, Adelaide, Australia; ^12^Sydney Children’s Hospital, Sydney, Australia; ^13^Royal Children’s Hospital Brisbane, Brisbane, Australia; ^14^Poole Hospital NHS Foundation Trust, Poole, UK; ^15^Salisbury Healthcare NHS Trust, Salisbury, UK

**Corresponding author:**

Professor John Warner

Department of Paediatrics

Imperial College London

Norfolk Place, London UK. W2 1PG

Tel: +44 207 594 3990 Fax: +44 207 594 3984

email: [j.o.warner@imperial.ac.uk](mailto:j.o.warner@imperial.ac.uk)

*Both authors contributed equally

**Conflict of interest**: The study was funded by Nutricia Research. MLKT is a member of the ANZ medical advisory boards for Danone Nutricia and Nestle Nutrition Institute; Global scientific advisory board for Danone Nutricia; and has received honoraria for presentations at symposia sponsored by Danone Nutricia and Nestle Nutrition Institute.  JOBH is chair of the Irish Food Allergy Network which receives unrestricted educational grants from Danone Nutricia and other manufacturers of infant formulae and has received honoraria for presentations at symposia sponsored by Danone Nutricia and other companies. JOW is a member of the global advisory board for Danone Nutricia and has received grants and honoraria for presentations at symposia sponsored by Danone Nutricia. The other authors declare no conflict of interest.

**†PATCH Study Team:** Christine Axelrad, Royal Children’s Hospital Melbourne; Suzan Jeffries, Imperial College London; Yvette Donald and Heather Barham, Poole Hospital; Jenny Brown and Rita Wickenden, Salisbury District Hospital; Teresa Barnes, Gold Coast Hospital; Simone Taylor, Royal Children’s Hospital Brisbane; Susan Smith, Sydney Children’s Hospital; Natalie Thomas, Women’s and Children’s Hospital Adelaide; Anne Goh, Wong Anng Anng and Christy Cao Yu Hua, KK Women’s and Children Hospital Singapore; Deirdre Daly, Sinead Lafford and Claire Cullinane, Cork University Hospital; Jacques Bindels, Liandre van der Merwe, Dineke Klaassen, Sophie Swinkels and Karen Knipping, Nutricia Research.

**Keywords:**

Partial hydrolysate, infant formula, dietary intervention, oligosaccharides, randomised controlled trial, eczema, immune system, prebiotics

**Table S1**. Effect of the intervention on incidence of AD, in per protocol analysis

|  | **Control** | **Active** | **Effect Measure**  **(95% CI)** | **Adjusted effect**  **(95% CI) Model 1** | **Adjusted effect**  **(95% CI) Model 2** |
| --- | --- | --- | --- | --- | --- |
| Eczema by 12 months | 56/173 (32.4%) | 45/142 (31.7%) | OR 1.03  (0.63, 1.71) | OR 1.01  (0.60, 1.69) | OR 1.03  (0.63, 1.71) |
| Eczema by 18 months | 58/171 (33.9%) | 52/142 (36.6%) | OR 0.90  (0.55, 1.48) | OR 0.86  (0.52, 1.43) | OR 0.90  (0.55, 1.48) |

All analyses were adjusted for stratification variables study centre and maternal allergic history. In addition, Model 1 adjusted for all predefined covariates (i.e. sex, ethnicity, mode of birth, pet exposure, presence of siblings, birth weight) and Model 2 adjusted for all covariates that were significant in Model 1 (none at 12 and 18 months).

**Table S2.** Baseline characteristics of all randomised subjects and the subgroup selected for PBMC analysis

|  | **Control [ASR]**  **(N=431)** | **Control [subgroup]**  **(N=46)** | **Active [ASR]**  **(N=432)** | **Active [subgroup]**  **(N=39)** |
| --- | --- | --- | --- | --- |
| Maternal age (years) | 30.0 (6.5) | 30.4 (6.8) | 29.3 (7.0) | 29.2 (6.4) |
| Maternal tertiary education | 134 (31%) | 10 (22%) | 127 (29%) | 10 (26%) |
| Gestational Age (weeks) | 38.9 (1.3) | 38.9 (1.1) | 39.0 (1.3) | 38.7 (1.2) |
| Male Sex | 209 (48%) | 17 (37%) | 238 (55%) | 21 (54%) |
| Mean (SD) birth weight (g) | 3306 (463) | 3216 (483) | 3302 (486) | 3330 (472) |
| Both parents allergic  Only mother allergic  Only father allergic | 90 (21%)  240 (56%)  99 (23%) | 9 (20%)  9 (20%)  28 (60%) | 79 (18%)  250 (58%)  102 (24%) | 9 (23%)  9 (23%)  21 (54%) |
| Caucasian ethnicity  Asian ethnicity  Other ethnicity | 157 (38%)  240 (58%)  14 (4%) | 6 (13%)  38 (83%)  2 (4%) | 156 (38%)  237 (58%)  17 (4%) | 12 (31%)  25 (64%)  2 (5%) |
| Vaginal delivery  Instrumental delivery  Caesarean delivery | 267 (62%)  32 (7%)  132 (31%) | 32 (70%)  4 (9%)  10 (21%) | 279 (65%)  37 (9%)  116 (27%) | 23 (59%)  3 (8%)  13 (33%) |
| Pet at home | 130 (30%) | 7 (15%) | 138 (32%) | 8 (21%) |
| At least one sibling | 235 (55%) | 29 (63%) | 231 (53%) | 20 (51%) |
| Australia  Ireland  Singapore  UK | 85 (20%)  27 (6%)  237 (55%)  82 (19%) | 7 (15%)  0 (0%)  38 (83%)  1 (2%) | 85 (20%)  26 (6%)  237 (55%)  84 (19%) | 9 (23%)  0 (0%)  25 (64%)  5 (13%) |

ASR = All subjects randomised; PBMC = peripheral blood mononuclear cell; SD = standard deviation

**Table S3**. Adverse Events from enrolment to 18 months follow up – specific categories

|  | Control  (n=431) | | Active  (n=432) | | Breastfed  (n=184) |
| --- | --- | --- | --- | --- | --- |
|  | **Related** | **Total** | **Related** | **Total** | **Total** |
|  | **n (%)** | **n (%)** | **n (%)** | **n (%)** | **n (%)** |
| Gastrointestinal disorders | 65 (15.1) | 187 (43.4) | 69 (16) | 195 (45.1) | 97 (52.7) |
| Immune system disorders | 12 (2.8) | 55 (12.8) | 15 (3.5) | 43 (10) | 29 (15.8) |
| Infections and infestations | 20 (4.6) | 333 (77.3) | 18 (4.2) | 310 (71.8) | 120 (65.2) |
| Skin and subcutaneous tissue disorders | 97 (22.5) | 238 (55.2) | 74 (17.1) | 244 (56.5) | 120 (65.2) |
| Respiratory, thoracic, mediastinal disorders | 10 (2.3) | 132 (30.6) | 14 (3.2) | 138 (31.9) | 58 (31.5) |
| Blood and lymphatic system disorders | 0 (0) | 5 (1.2) | 0 (0) | 3 (0.7) | 2 (1.1) |
| Cardiac disorders | 0 (0) | 4 (0.9) | 0 (0) | 6 (1.4) | 1 (0.5) |
| Congenital, familial and genetic disorders | 0 (0) | 46 (10.7) | 0 (0) | 53 (12.3) | 15 (8.2) |
| Ear and labyrinth disorders | 1 (0.2) | 5 (1.2) | 0 (0) | 8 (1.9) | 7 (3.8) |
| Endocrine disorders | 0 (0) | 1 (0.2) | 0 (0) | 0 (0) | 0 (0) |
| Eye disorders | 4 (0.9) | 57 (13.2) | 0 (0) | 48 (11.1) | 18 (9.8) |
| General disorders, administration site conditions | 8 (1.9) | 125 (29) | 5 (1.2) | 124 (28.7) | 41 (22.3) |
| Hepatobiliary disorders | 0 (0) | 2 (0.5) | 0 (0) | 3 (0.7) | 1 (0.5) |
| Injury, poisoning, procedural complications | 1 (0.2) | 39 (9) | 0 (0) | 47 (10.9) | 19 (10.3) |
| Investigations | 1 (0.2) | 43 (10) | 3 (0.7) | 45 (10.4) | 16 (8.7) |
| Metabolism and nutrition disorders | 4 (0.9) | 17 (3.9) | 4 (0.9) | 26 (6) | 6 (3.3) |
| Musculoskeletal, connective tissue disorders | 2 (0.5) | 13 (3) | 4 (0.9) | 19 (4.4) | 5 (2.7) |
| Neoplasms, malignant and unspecified (including cysts and polyps) | 0 (0) | 5 (1.2) | 0 (0) | 6 (1.4) | 2 (1.1) |
| Nervous system disorders | 0 (0) | 13 (3) | 0 (0) | 11 (2.5) | 5 (2.7) |
| Pregnancy, puerperium, perinatal conditions | 1 (0.2) | 56 (13) | 2 (0.5) | 53 (12.3) | 10 (5.4) |
| Psychiatric disorders | 0 (0) | 9 (2.1) | 6 (1.4) | 14 (3.2) | 9 (4.9) |
| Renal and urinary disorders | 0 (0) | 2 (0.5) | 0 (0) | 2 (0.5) | 1 (0.5) |
| Reproductive system and breast disorders | 0 (0) | 6 (1.4) | 0 (0) | 8 (1.9) | 2 (1.1) |
| Social circumstances | 0 (0) | 2 (0.5) | 0 (0) | 1 (0.2) | 0 (0) |
| Surgical and medical circumstances | 0 (0) | 6 (1.4) | 0 (0) | 7 (1.6) | 8 (4.3) |
| Vascular disorders | 0 (0) | 1 (0.2) | 0 (0) | 4 (0.9) | 1 (0.5) |
| Unknown categorisation | 0 (0) | 2 (0.5) | 0 (0) | 2 (0.5) | 2 (1.1) |

**Table S4**. Serious Adverse Events from enrolment to 18 months follow up – specific categories

|  | Control  (n=431) | | Active  (n=432) | | Breastfed  (n=184) |
| --- | --- | --- | --- | --- | --- |
|  | **Related** | **Total** | **Related** | **Total** | **Total** |
|  | **n**  **(%)** | **n**  **(%)** | **n**  **(%)** | **n**  **(%)** | **n**  **(%)** |
| Gastrointestinal disorders | 0 (0) | 3 (0.7) | 0 (0) | 8 (1.9) | 3 (1.6) |
| Immune system disorders | 0 (0) | 1 (0.2) | 1 (0.2) | 1 (0.2) | 0 (0) |
| Infections and infestations | 0 (0) | 52 (12.1) | 3 (0.7) | 58 (13.4) | 7 (3.8) |
| Skin and subcutaneous tissue disorders | 0 (0) | 1 (0.2) | 1 (0.2) | 3 (0.7) | 1 (0.5) |
| Respiratory, thoracic, mediastinal disorders | 1 (0.2) | 8 (1.9) | 1 (0.2) | 5 (1.2) | 3 (1.6) |
| Blood and lymphatic system disorders | 0 (0) | 1 (0.2) | 0 (0) | 0 (0) | 0 (0) |
| Cardiac disorders | 0 (0) | 0 (0) | 0 (0) | 1 (0.2) | 1 (0.5) |
| Congenital, familial and genetic disorders | 0 (0) | 4 (0.9) | 0 (0) | 5 (1.2) | 0 (0) |
| Ear and labyrinth disorders | 0 (0) | 0 (0) | 0 (0) | 0 (0) | 0 (0) |
| Endocrine disorders | 0 (0) | 1 (0.2) | 0 (0) | 0 (0) | 0 (0) |
| Eye disorders | 0 (0) | 0 (0) | 0 (0) | 0 (0) | 0 (0) |
| General disorders, administration site conditions | 1 (0.2) | 8 (1.9) | 1 (0.2) | 5 (1.2) | 1 (0.5) |
| Hepatobiliary disorders | 0 (0) | 0 (0) | 0 (0) | 0 (0) | 1 (0.5) |
| Injury, poisoning, procedural complications | 0 (0) | 7 (1.6) | 0 (0) | 3 (0.7) | 4 (2.2) |
| Investigations | 0 (0) | 3 (0.7) | 0 (0) | 0 (0) | 2 (1.1) |
| Metabolism and nutrition disorders | 0 (0) | 1 (0.2) | 1 (0.2) | 4 (0.9) | 1 (0.5) |
| Musculoskeletal, connective tissue disorders | 0 (0) | 0 (0) | 0 (0) | 1 (0.2) | 0 (0) |
| Neoplasms, malignant and unspecified (including cysts and polyps) | 0 (0) | 0 (0) | 0 (0) | 0 (0) | 0 (0) |
| Nervous system disorders | 0 (0) | 4 (0.9) | 0 (0) | 7 (1.6) | 1 (0.5) |
| Pregnancy, puerperium, perinatal conditions | 0 (0) | 11 (2.6) | 0 (0) | 7 (1.6) | 0 (0) |
| Psychiatric disorders | 0 (0) | 2 (0.5) | 0 (0) | 0 (0) | 0 (0) |
| Renal and urinary disorders | 0 (0) | 0 (0) | 0 (0) | 1 (0.2) | 0 (0) |
| Reproductive system and breast disorders | 0 (0) | 1 (0.2) | 0 (0) | 0 (0) | 0 (0) |
| Social circumstances | 0 (0) | 1 (0.2) | 0 (0) | 0 (0) | 0 (0) |
| Surgical and medical circumstances | 0 (0) | 1 (0.2) | 0 (0) | 0 (0) | 1 (0.5) |
| Vascular disorders | 0 (0) | 0 (0) | 0 (0) | 1 (0.2) | 0 (0) |
| Unknown categorisation | 0 (0) | 0 (0) | 0 (0) | 0 (0) | 0 (0) |

**Table S5**. Gastrointestinal symptoms in study participants

|  |  | **12 week visit** | | | **26 week visit** | | |
| --- | --- | --- | --- | --- | --- | --- | --- |
|  | **Frequency** | **Control**  **(n=332)** | **Active**  **(n=306)** | **P** | **Control**  **(n=322)** | **Active**  **(n=294)** | **P** |
| **Vomiting** | Occasional | 77 (23.2%) | 58 (19.0%) | 0.42 | 56 (17.4%) | 47 (16.0%) | 0.30 |
|  | Frequent | 13 (3.9%) | 12 (3.9%) |  | 13 (4.0%) | 6 (2.0%) |  |
| **Burping** | Occasional | 133 (41.3%) | 138 (45.1%) | 0.048 | 139 (43.2%) | 133 (45.2%) | 0.82 |
|  | Frequent | 84 (26.1%) | 53 (17.3%) |  | 44 (13.7%) | 36 (12.2%) |  |
| **Flatulence** | Occasional | 132 (41.0%) | 133 (43.5%) | 0.25 | 187 (58.1%) | 168 (57.1%) | 0.82 |
|  | Frequent | 188 (58.4%) | 156 (51.0%) |  | 92 (28.6%) | 90 (30.6%) |  |
| **Diarrhoea** | Occasional | 22 (6.8%) | 26 (8.5%) | 0.38 | 27 (8.4%) | 42 (14.3%) | 0.07 |
|  | Frequent | 4 (1.2%) | 7 (2.3%) |  | 3 (0.9%) | 3 (1.0%) |  |
| **Constipation** | Occasional | 18 (5.6%) | 11 (3.6%) | 0.56 | 14 (4.3%) | 13 (4.4%) | 1.00 |
|  | Frequent | 4 (1.2%) | 3 (1.0%) |  | 1 (0.3%) | 1 (0.3%) |  |
| **Nappy rashes** | Occasional | 61 (18.9%) | 64 (20.9%) | 0.26 | 93 (28.9%) | 83 (28.2%) | 0.98 |
|  | Frequent | 5 (1.6% | 1 (0.3%) |  | 4 (1.2%) | 4 (1.4%) |  |
| **Cramps (colic)** | Occasional | 78 (24.2%) | 63 (20.6%) | 0.64 | 26 (8.1%) | 26 (8.8%) | 0.71 |
|  | Frequent | 8 (2.5%) | 9 (2.9%) |  | 3 (0.9%) | 1 (0.3%) |  |
| **Possetting** | Occasional | 213 (66.1%) | 206 (67.3%) | 0.57 | 152 (47.2%) | 145 (49.3%) | 0.67 |
|  | Frequent | 43 (13.4%0 | 32 (10.5%) |  | 24 (7.5%) | 17 (5.8%) |  |

Statistical comparisons were made using Chi-Square, or where expected values were <5 using Fisher’s Exact test.

**Table S6**. Stool frequency and consistency in study participants

|  | **Control** | **Active** | **P** | **MD (95%CI)** | **Breastfed** |
| --- | --- | --- | --- | --- | --- |
| Frequency 4 weeks | 2.77 (2.34) | 3.45 (2.48) | <0.001 | 0.68 (0.31, 1.05) | 5.26 (2.67) |
| Frequency 12 weeks | 1.65 (1.45) | 1.73 (1.39) | 0.48 | 0.08 (-0.14, 0.30) | 2.65 (2.16) |
| Frequency 6 months | 1.43 (0.87) | 1.60 (1.01) | 0.03 | 0.17 (0.02, 0.32) | 1.63 (1.25) |
| Frequency 12 months | 1.69 (0.91) | 1.58 (0.84) | 0.13 | -0.11 (-0.25, 0.03) | 1.92 (0.99) |
| Watery at 4 weeks  Soft at 4 weeks  Dry/hard at 4 weeks | 127 (37.1%)  209 (61.1%)  6 (1.8%) | 130 (40.4%)  190 (59.0%)  2 (0.6%) | 0.27 |  | 151 (66.5%)  76 (33.5%)  0 (0%) |
| Watery at 12 weeks  Soft at 12 weeks  Dry/hard at 12 weeks | 113 (34.0%)  216 (65.1%)  3 (0.9%) | 130 (42.2%)  178 (57.8%)  0 (0%) | 0.020 |  | 143 (96.0%)  6 (4.0%)  0 (0%) |
| Watery at 6 months  Soft at 6 months  Dry/hard at 6 months | 70 (21.8%)  241 (75.1%)  10 (3.1%) | 77 (26.2%)  214 (72.8%)  3 (1.0%) | 0.10 |  | 88 (63.3%)  45 (32.4%)  6 (4.3%) |
| Watery at 12 months  Soft at 12 months  Dry/hard at 12 months | 38 (12.3%)  255 (82.3%)  17 (5.5%) | 20 (7.1%)  234 (83.6%)  26 (9.3%) | 0.032 |  | 36 (27.1%)  94 (70.7%)  3 (2.3%) |

Data shown are parent-reported mean (sd) stool frequency in the 4 week period prior to 4 week, 6 month and 12 month assessments ( ‘early introduction subgroup’). Data were available for 342 control, 322 active, 184 breastfed at 4 weeks; 332, 308 and 149 at 12 weeks; 322, 294, 139 at 6 months; 310, 280 and 132 at 12 months. Statistical comparisons were made using t-test for continuous data, and Mantel-Haenszel Chi-Square test for categorical data.

**Table S7**. Growth measures in study participants randomised before 4 weeks age or not randomised (‘breastfed’).

|  | **Control (n=383)** | **n** | **Active (n=375)** | **n** | **P** | **MD (95%CI)** | **Breastfed (n=184)** |
| --- | --- | --- | --- | --- | --- | --- | --- |
| **Mean (sd) weight (g)** | | | |  |  |  |  |
| Birth | 3274 (462) | 382 | 3272 (481) | 375 | 0.96 | -2 (-69, 66) | 3541 (439) |
| 4 weeks | 4204 (514) | 341 | 4235 (547) | 320 | 0.45 | 31 (-50, 112) | 4348 (592) |
| 12 weeks | 5862 (713) | 331 | 5948 (725) | 308 | 0.13 | 86 (-26, 198) | 5934 (790) |
| 6 months | 7747 (945) | 322 | 7747 (928) | 292 | 1.00 | 0 (-149, 149) | 7665 (989) |
| 12 months | 9631 (1214) | 308 | 9651 (1250) | 278 | 0.84 | 20 (-180, 220) | 9669 (1115) |
| 18 months | 11003 (1486) | 289 | 10976 (1446) | 260 | 0.83 | -27 (-273, 219) | 11395 (1353) |
| **Mean (sd) length (cm)** | | | |  |  |  |  |
| Birth | 49.3 (2.7) | 342 | 49.5 (2.5) | 336 | 0.32 | 0.2 (-0.2, 0.6) | 51.7 (2.9) |
| 4 weeks | 53.3 (2.4) | 340 | 53.7 (2.4) | 319 | 0.03 | 0.4 (0.0, 0.8) | 54.7 (2.5) |
| 12 weeks | 59.6 (2.4) | 326 | 60.2 (2.6) | 307 | 0.003 | 0.6 (0.2, 1.0) | 60.8 (2.9) |
| 6 months | 66.7 (2.7) | 321 | 66.9 (2.9) | 292 | 0.38 | 0.2 (-0.2, 0.6) | 66.9 (3.2) |
| 12 months | 74.6 (3.1) | 307 | 74.9 (3.2) | 278 | 0.25 | 0.3 (-0.2, 0.8) | 75.1 (3.1) |
| 18 months | 81.1 (3.5) | 288 | 81.4 (3.7) | 260 | 0.33 | 0.3 (-0.3, 0.9) | 82.0 (3.6) |
| **Mean (sd) head circumference (cm)** | | | |  |  |  |  |
| Birth | 33.9 (1.6) | 354 | 33.9 (1.6) | 353 | 1.00 | 0.0 (-0.2, 0.2) | 34.8 (1.5) |
| 4 weeks | 36.8 (1.2) | 339 | 37.0 (1.3) | 319 | 0.04 | 0.2 (-0.0, 0.4) | 37.5 (1.4) |
| 12 weeks | 39.7 (1.4) | 329 | 40.1 (1.4) | 309 | 0.0003 | 0.4 (0.2, 0.6) | 40.7 (1.4) |
| 6 months | 43.0 (1.4) | 317 | 43.1 (1.5) | 290 | 0.40 | 0.1 (-0.1, 0.3) | 43.7 (1.6) |
| 12 months | 45.9 (1.6) | 306 | 46.0 (1.7) | 277 | 0.46 | 0.1 (-0.2, 0.4) | 46.6 (1.5) |
| 18 months | 47.4 (1.6) | 286 | 47.6 (1.6) | 261 | 0.14 | 0.2 (-0.1, 0.5) | 48.2 (1.7) |

Statistical comparisons were made using t-test
